# Missouri

**Published:** 1897-05

**Authors:** 


*Missouri. Bulletin No.* 37 of the State Experiment Station records the results of some interesting experiments bearing upon the transmission of Texas-fever by the ticks. Ticks hatched in the laboratory and placed upon some Missouri short-horn cows in lots free from any possible infection resulted in fatal cases of Texas-fever in from thirteen to eighteen days. Some dipped cattle from the Texas Experiment Station (West Virginia mineral oil being used) were sent to Missouri and placed among some susceptible short-horn cattle from August 13th to November 1st without the development of any symptoms of Texas-fever. Some inoculation experiments have been made which promise very well and which may prove a thorough and inexpensive solution of this problem.

